# Advancement in pleura effusion diagnosis: a systematic review and meta-analysis of point-of-care ultrasound versus radiographic thoracic imaging

**DOI:** 10.1186/s13089-023-00356-z

**Published:** 2024-01-23

**Authors:** Hany A. Zaki, Bilal Albaroudi, Eman E. Shaban, Ahmed Shaban, Mohamed Elgassim, Nood Dhafi Almarri, Kaleem Basharat, Aftab Mohammad Azad

**Affiliations:** 1 Department of Emergency Medicine, Hamad Medical Corporation, Doha, Qatar; 2 Department of Cardiology, Al Jufairi Diagnosis and Treatment, MOH, Doha, Qatar; 3 Department of Internal Medicine, Mansoura General Hospital, Mansoura, Egypt; 4 Hamad Medical Corporation, Collège of Medicine QU and Weil Cornell Medical College, Doha, Qatar

**Keywords:** Pleural effusion, Diagnostic imaging, Emergency medicine, Meta-analysis, Point-of-care systems, Ultrasound, Chest X-ray, Sensitivity, Specificity, Systematic review

## Abstract

**Background:**

Pleural effusion is a fluid buildup in the pleural space that mostly result from congestive heart failure, bacterial pneumonia, malignancy, and pulmonary embolism. The diagnosis of this condition can be challenging as it presents symptoms that may overlap with other conditions; therefore, imaging diagnostic tools such as chest x-ray/radiograph (CXR), point-of-care ultrasound (POCUS), and computed tomography (CT) have been employed to make an accurate diagnosis. Although POCUS has high diagnostic accuracy, it is yet to be considered a first-line diagnostic tool as most physicians use radiography. Therefore, the current meta-analysis was designed to compare POCUS to chest radiography.

**Methods:**

n extended search for studies related to our topic was done on five electronic databases, including PubMed, Medline, Embase, Scopus, and Google Scholar. A quality assessment using the Quality Assessment of Diagnostic Accuracy Studies tool (QUADAS-2) was performed on all eligible articles obtained from the databases. Moreover, the diagnostic accuracy of POCUS and CXR was performed using STATA 16 software.

**Results:**

Our search yielded 1642 articles, of which only 18 were eligible for inclusion and analysis. The pooled analysis showed that POCUS had a higher diagnostic accuracy compared to CXR (94.54% (95% CI 91.74–97.34) vs. 67.68% (95% CI 58.29–77.08) and 97.88% (95% CI 95.77–99.99) vs. 85.30% (95% CI 80.06–90.54) sensitivity and specificity, respectively). A subgroup analysis based on the position of patients during examinations showed that POCUS carried out in supine and upright positions had higher specificity than other POCUS positions (99%). In comparison, lateral decubitus CXR had higher sensitivity (96%) and specificity (99%) than the other CXR positions. Further subgroup analyses demonstrated that CXR had higher specificity in studies that included more than 100 patients (92.74% (95% CI 85.41–100). Moreover, CXR tends to have a higher diagnostic accuracy when other CXR positions are used as reference tests (93.38% (95% CI 86.30–100) and 98.51% (95% CI 94.65–100) sensitivity and specificity, respectively).

**Conclusion:**

POCUS as an imaging modality has higher diagnostic accuracy than CXR in detecting pleural effusion. Moreover, the accuracy is still high even when performed by physicians with less POCUS training. Therefore, we suggest it is considered a first-line imaging tool for diagnosing pleural effusion at the patients’ bedside.

## Introduction

Pleural effusion is a fluid buildup in the pleural space that affects approximately 320 persons out of every 100,000 in developed nations and at least 1.5 million people in the United States annually [1, 2]. The majority of these cases are caused by congestive heart failure, bacterial pneumonia, malignancy, or pulmonary embolism. Research suggests that over two-thirds of malignant pleural effusions occur in women, notably those with breast and gynecologic malignancies [3, 4]. Similarly, pleural effusions caused by systemic lupus erythematosus are more frequent in women than in males [4]. However, in the United States alone, pleural effusions resulting from malignant mesothelioma are more likely to manifest in males owing to increased occupational asbestos exposure [4]. Furthermore, pleural effusion mostly occurs in adult patients. However, it is becoming increasingly prevalent in children as a result of underlying pneumonia [5]. Pleural effusion in fetuses has also been documented, and in certain scenarios, it may be managed before birth [6].

The diagnosis of pleural effusion can be very challenging as it presents symptoms that may overlap with conditions such as pneumonia, pulmonary embolism, acute coronary syndrome, pneumothorax, chromonic obstructive pulmonary disease, heart failure, and pulmonary edema. Therefore, diagnostic tools are vital as they aid healthcare professionals make accurate diagnosis. Various imaging tests, including chest x-ray/radiograph (CXR), ultrasound, and computerized tomography (CT), have been adopted in detecting pleural effusion. Traditionally, CXR was considered a first-line imaging tool for pleural effusions. However, evidence reveals that upright CXR may miss a considerable percentage of pleural effusions. Brixey and colleagues found that upright CXR missed as much as 10% of parapneumonic effusions that were substantial enough to suggest the need for drainage [7]. Moreover, other researchers have reported that supine anterior–posterior CXR might miss a large number of pleural effusions compared to chest CT, ultrasound, and lateral decubitus radiographs [8–10].

On the other hand, point-of-care ultrasound (POCUS) has gained popularity in diagnosing pleural effusions because it aids healthcare professionals to gather and analyze images at the bedside and make quick decisions. Moreover, data pooled from previous studies have shown that it has a very high sensitivity and specificity [11]. However, it is yet to be considered a first-line diagnostic tool for pleural effusion as most physicians use radiography. Therefore, the current meta-analysis was designed to compare POCUS to chest radiography and make a clear recommendation for healthcare professionals.

## Methods

### Protocol and registration

This systematic review and meta-analysis was conducted in accordance with the Preferred Reporting Items for Systematic Reviews and Meta-Analyses (PRISMA) guiding principles and protocol registered on PROSPERO article (CRD42023420515)**.**

### Eligibility criteria

Two independent reviewers derived a set of conditions to include and exclude articles in the present study. In case of discrepancies during this process, the reviewers engaged in constructive debates. The criteria used to select studies for inclusion were as follows:

1. Randomized trials or observational studies written and published in English. This criterion assisted us in evading the literal translation of scientific terminologies, which would have hampered our scientific goal.
2. Studies that directly compared POCUS to chest x-ray or individually assessed the role of these imaging tests in the diagnosis of pleural effusions.
3. Studies reporting at least one of the following outcomes: sensitivity, specificity, or true positives, true negatives, false negatives, and false positives.

Conversely, studies were regarded ineligible for inclusion due to the following reasons.

1. Studies that were designed as either systematic reviews, meta-analyses, abstracts without full articles, case reports and series, letters to the editor, guidelines, or recommendations.
2. Studies that evaluated the accuracy of either POCUS or CXR in diagnosing underlying diseases associated with pleural effusion or other conditions.
3. Studies that integrated POCUS or CXR with other diagnostic tools when evaluating pleural effusion.

### Literature search

Two reviewers independently explored five electronic databases (PubMed, Medline, Embase, Scopus, and Google Scholar) for papers related to the topic at hand. To ease the search on these databases, the reviewers employed the Boolean operators “AND” and “OR” to integrate keywords and produce well-defined mesh phrases. These mesh phrases were as follows: (“Point of care ultrasound” OR “POCUS” OR “bedside ultrasound” OR “sonography”) AND (“Chest x-rays” OR “Chest Radiography” OR “Radiology”) AND (“pleural effusion” OR “parapneumonic effusion” OR “effusion” OR “Pleural free fluid”). The reviewers also screened reference lists of articles from these databases for additional studies and excluded all close or exact duplicates and grey literature to improve the scientific purpose of our study.

### Quality appraisal

Our research was structured as a diagnostic review; therefore, two experienced reviewers were asked to independently evaluate methodological quality using the Quality Assessment of Diagnostic Accuracy Studies (QUADAS-2) tool provided in the Review Manager software (RevMan 5.4.1). Using this framework, the reviewers derived various signaling questions to judge the risk of bias, applicability, and concerns. Any discrepancies during this process were resolved by consulting a third reviewer.

### Data extraction

The two reviewers assigned for data extraction independently gathered and assembled relevant data in a tabular manner (Table 1). The data extracted included; Author ID (surname of the first author and year the study was published), study design, location of the study (Country), characteristics of the study population (sample size, gender distribution, and mean/median age), reference tests, ultrasound and x-ray machines used, operators, and main outcomes. The main outcomes were specificity, sensitivity, false negatives, and false positives. In case of disagreements, the two reviewers resolved their issues through constructive dialogues or by sorting the opinion of a third reviewer. Moreover, web-based programs were used to calculate either sensitivity or specificity in studies where data were not presented.

**Table 1:** Study Characteristics

Author ID	Study design	Location	Participants’ characteristics	Reference tests	POCUS		Chest x-ray		Main Outcomes
					Machine	Position	Machine	Position	
Elmahalawy et al. 2016 [12]	A prospective randomized single-group observational study	Egypt	130 patients (84 males and 46 females; mean age: 43.23 ± 12.62 years)	CT scans	Micro convex 5–9 MHz transducer	Supine	Portable x-ray machine	Upright	CXR was 70% sensitive and 90% specific in diagnosing pleural effusion, while POCUS was 94% sensitive and 96% specific
Graven et al. 2015 [13]	Prospective single-center observational study	Norway	59 patients (20 females and 39 males; mean age: 67 (35–86) years)	Echocardiography performed by 4 cardiologists	PSID Vscan (version 1.2) with an adjustable bandwidth of 1.7–3.8 MHz	Lateral decubitus	NR	Upright and lateral	The US exam detected pleural effusion with a 98% sensitivity and 70% specificity, while CXR had a 40% sensitivity and 78% specificity
Rocco et al. 2008 [14]	Prospective clinical study	Italy	15 patients (10 males and 5 females; mean age: 42 ± 14 years)	CT scans	Aloka SSD 1700 with a 3.5 MHz convex probe	Supine	Portable radiograph Siemens Mobilett II with a high voltage (80–90 kV)	Supine	CXR was less sensitive than the US in diagnosing PE (42 vs. 94%); however, the specificity was relatively similar (97 vs. 99%, respectively)
Mohamed.,2018 [15]	Comparative cross-sectional study	Egypt	60 patients (38 male and 22 females; mean age: 53.83 ± 14.63 years)	CT scans	YD-9000 A and Fukuda denshi (UF-400AX) with 3.5–5 MHz and 5–8 MHz probes	Supine	Siemens portable X-ray machine	Supine	CXR had a poor diagnostic accuracy for pleural effusion than chest US (76.2 vs. 100% sensitivity and 70.6 vs. 100% specificity, respectively.)
Rozycki et al. 2001 [16]	Observational study	United States	47 patients (41 males and 6 females; mean age: 44.4 ± 17.9 years)	CXR and CT scans	B&K Panther 2002 US scanner and a 3.5MHz transducer	Supine	NR	NR	The US exams yielded an 83.6% sensitivity, 100% specificity, and 94% accuracy for detecting pleural effusion
Xirouchaki et al. 2011 [17]	Prospective Study	Greece	42 patients (34 male and 8 females; mean age: 57.1 ± 21.5 years)	CT scans	Micro-convex 5–9 MHz transducer	Supine	Portable X-ray machine (Siemens Polymobile, Erlangen, Germany)	Supine	The sensitivity and specificity of CXR to diagnose pleural effusion were poor compared to POCUS (65 vs. 100% and 81 vs. 100%, respectively)
Walsh et al. 2021 [18]	A prospective multicenter study	Canada and the United States	34 patients (16 male and 18 female)	Chest radiography by radiologists	Low-frequency transducer (2–5 MHz in Canada and 1–5 MHz in the United States)	Upright and supine	NR	NR	US exams carried out in upright and supine positions had very high accuracies in diagnosing pleural effusion (92% and 98% sensitivity and 94.4% specificity, respectively)
Schieder et al. 2012 [19]	Observational study	Germany	24 patients (14 male and 10 females; median age: 65 (42–91) years)	High-end US	VScan with a plane 1.7–3.8 MHz transducer for 2D imaging and a 3.5-inch color LCD	Supine	Portable X-ray device (Mobilett XP ECO, Siemens Healthcare, Erlangen, Germany)	Supine	HCU had a higher accuracy for diagnosing pleural effusion than CXR (91 vs. 74% sensitivity and 100 vs. 31% specificity, respectively)
Ahmed et al. 2022 [20]	Cross-sectional Study	Egypt	40 patients (22 male and 18 females; mean age: 50.5 ± 17.1 years)	CT scans	A curved array probe operating at 5 MHz frequency	Supine and upright	NR	NR	Chest US was 100% sensitive and specific in diagnosing pleural effusion
Kocijancic et al. 2002 [21]	Case controlled Study	Slovenia	69 patients (51 men and 18 women; mean age: 57.1 years)	US or thoracentesis	NR	NR	140-kV Sire graph D3 unit (Siemens, Erlangen, Germany)	lateral decubitus	48 of the 52 patients showed positive results for pleural effusion on lateral decubitus radiographs (92% PPV)
Moller.,1984 [22]	Observational study	Sweden	100 patients (47 men and 53 women aged 17–94 years)	Oblique semi-supine CXR	NR	NR	NR	Lateral decubitus	38 true positives, 1 false positive, 5 false negatives, and 68 true negatives were recorded on the lateral decubitus views
Ruskin et al. 1987 [8]	Prospective study	United States	34 patients	Lateral decubitus CXR	NR	NR	40-inch target film	Supine	The sensitivity and specificity of supine CXR for detecting pleural effusion were 67% and 70%, respectively
Emamian et al. 1997 [10]	Comparative Study	Denmark	59 patients (28 men and 31 women; median age: 66 (16–93) years)	US	NR	NR	High kilovoltage (110–150) and portable grid with a 1/10 grid ratio	Supine	Supine CXR had an overall accuracy of 82% for detecting pleural effusion (82% sensitivity and 82% specificity)
Danish et al. 2019 [23]	Cross-sectional observational study	India	90 patients (67 males and 23 females; mean age: 47.66 ± 16.21 years)	CT scan	2–5 MHz curvilinear probe with SonoSite M-turbo portable USG machine	Supine	A portable device MobileArt eco MUX 10	supine	Compared to POCUS, CXR had a poor accuracy for detecting pleural effusion (47.5 vs. 92.9% and 71.8 vs. 100%, sensitivity, and specificity, respectively)
Mumtaz et al. 2017 [24]	Descriptive validation study	Pakistan	80 patients (57 males and 23 females)	CT scan	NR	Supine	NR	Supine	The overall diagnostic accuracy of US for detecting pleural effusion was higher than that of CXR (90 vs. 81.25%, respectively)
Kitazono et al. 2010 [9]	Retrospective study	United States	100 patients (60 male and 40 females; mean age 54.3 (14–91) years)	CT scan	NR	NR	Mobile x-ray unit (AMX-4 + , GE Healthcare)	Supine	CXR had an overall sensitivity and specificity of 66% and 89% for detecting pleural effusion
Lichtenstein et al. 2004 [25]	Prospective study	France	32 patients (mean age: 58 ± 15 years)	CT scan	Hitachi-405 and a micro convex 5MHz probe	Supine and lateral	AMX4 with high voltage (120–130kV)	Supine	POCUS exam had a higher diagnostic accuracy for detecting pleural effusion than CXR (92 vs. 39%, and 93 vs. 85%, sensitivity, and specificity, respectively)
Brixey et al. 2011 [7]	Retrospective study	United States	61 patients (34 males and 27 females; mean age: 56.8 ± 20.1 years)	CT scan	NR	NR	NR	Upright and lateral	The Upright PA CXR detected effusions with sensitivity and specificity of 82.1% and 81.3% The sensitivity and specificity of lateral CXR to identify effusions were 85.7% and 87.5%, respectively)

CXR: Chest x-ray/radiograph; POCUS: Point-of-care Ultrasound; US: Ultrasound; CT: Computerized tomography; HCU: Hand-carried ultrasound; NR: Not Reported; PA: Posteroanterior

### Data synthesis

STATA 16 statistical software was used to calculate the overall diagnostic accuracy of CXR and POCUS in detecting pleural effusion. To analyze the diagnostic accuracy, the sensitivity and specificity values with their 95% confidence intervals were pooled using the Der Simonian-Laird random effect model. Heterogeneity was also calculated using the I^2^ statistics, of which values between 0 and 49%, 50–70%, and 71–100% were regarded as low, moderate, and high, respectively. Moreover, we carried out subgroup analyses based on the position of the patients during the examinations, sample size, the country in which the study was carried out, reference test, POCUS level of training, POCUS machine, and CXR operator,

## Results

### Study selection

**Fig. 1:**
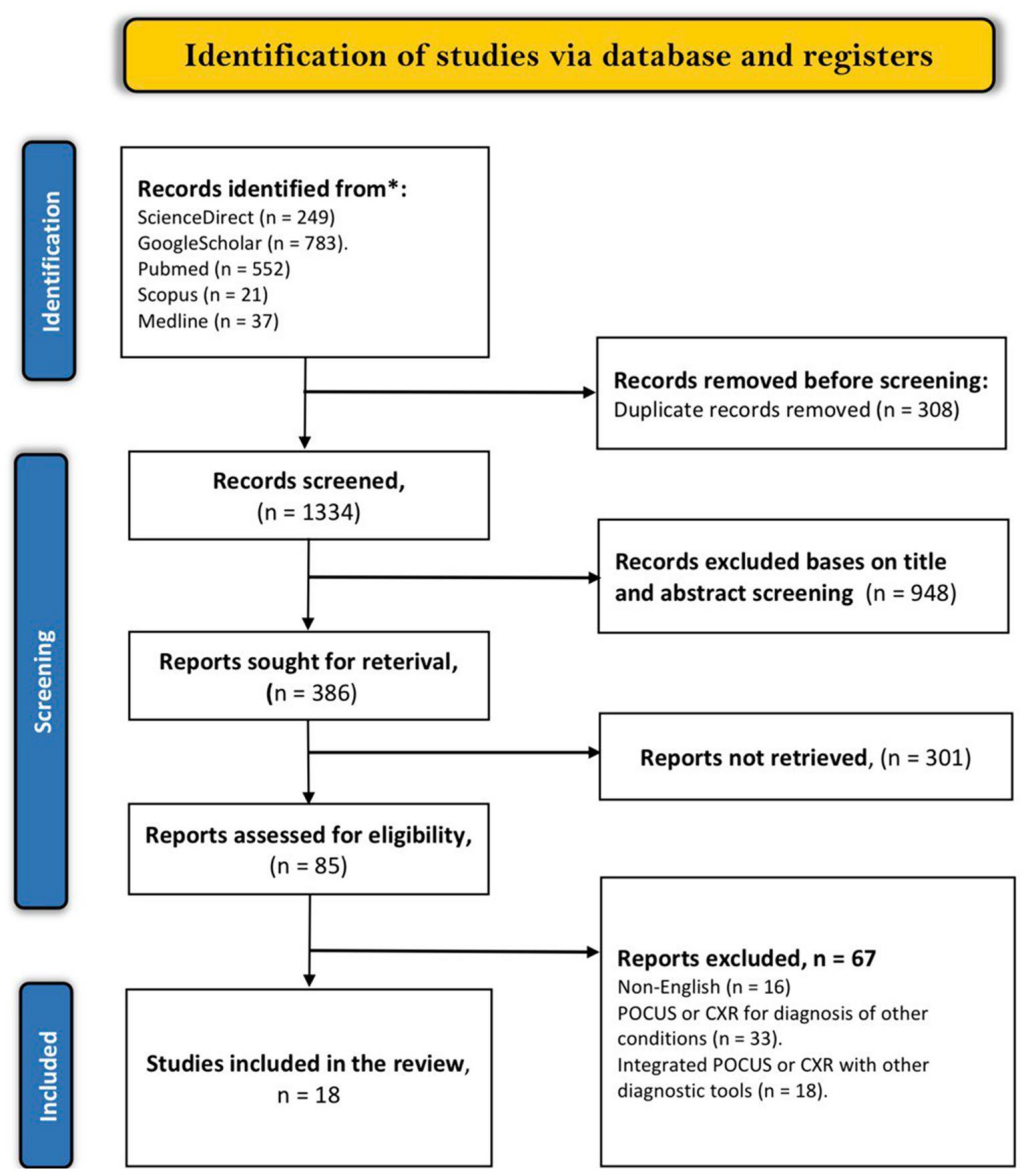
PRISMA flow diagram for study selection

After applying the mesh terms mentioned earlier on the electronic databases, 1642 articles were attained. A duplicate analysis of these articles revealed that 308 were either close or exact duplicates and were excluded. Titles and abstracts of the remaining articles were then screened, and 948 articles that did not meet the screening criteria were excluded. Out of the 386 remaining articles, 301 were not retrieved because they were either recommendation studies abstract without full articles, diagnostic algorithm studies, case reports, or systematic reviews. Finally, we included 18 articles [7–10, 12–25] (Table 1) as the other 67 articles were deemed ineligible due to the following reasons; 16 were published in other languages, 33 evaluated the accuracy of either POCUS or chest x-rays in the diagnosis of underlying diseases associated with the pleural effusion or other conditions and 18 articles integrated POCUS or CXR with other diagnostic tools when evaluating pleural effusion. The full selection criteria is summarized in PRISMA flow diagram below (Fig. 1).

### Quality assessment results

**Fig. 2:**
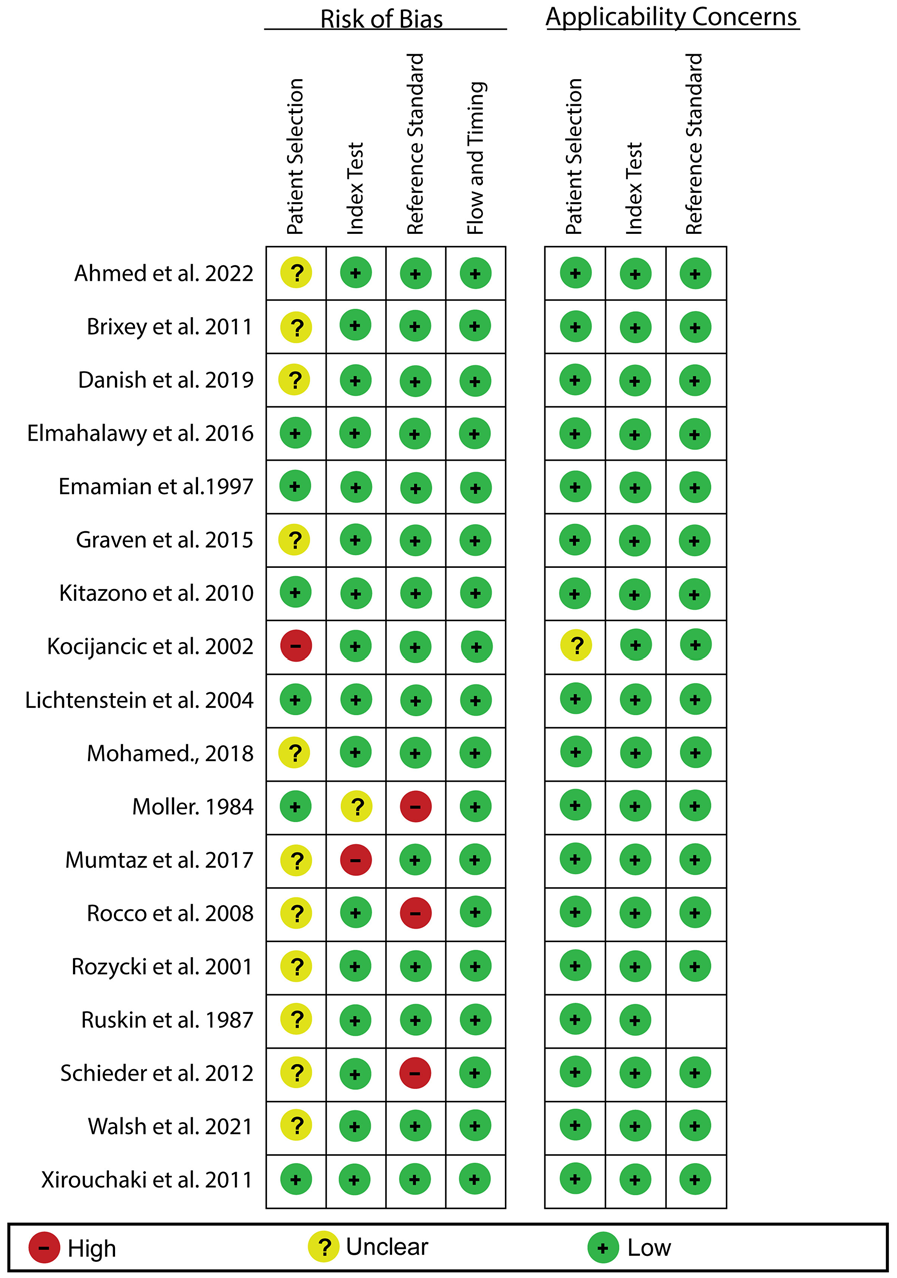
QUADAS-2 risk of bias summary

The risk of bias assessment is summarized in Fig. 2 below. The overall assessment using the QUADAS-2 tool has shown that all the studies included in our analysis have good methodological quality as they satisfied at least 4 of the 7 assessment criteria. In regard with patient selection, our evaluation revealed that most of the studies had an unclear risk bias because they did not specify the sampling method or used a convenience sampling. However, one of the studies showed a high risk of bias because it used a case control study design. Similarly, one study showed a high risk of bias based on the index test. This risk of bias was associated with the fact the radiologist who interpreted the reference test results (CT scan) also interpreted results of the index test (CXR); therefore, blinding of this interpreter to the index results was not possible. Moreover, our assessment revealed that the reference tests of three articles introduced a high a risk of bias to our analysis. The bias in these studies was because they used reference tests that were unlikely to classify pleural effusion correctly.

### Diagnostic performance of POCUS

**Fig. 3:**
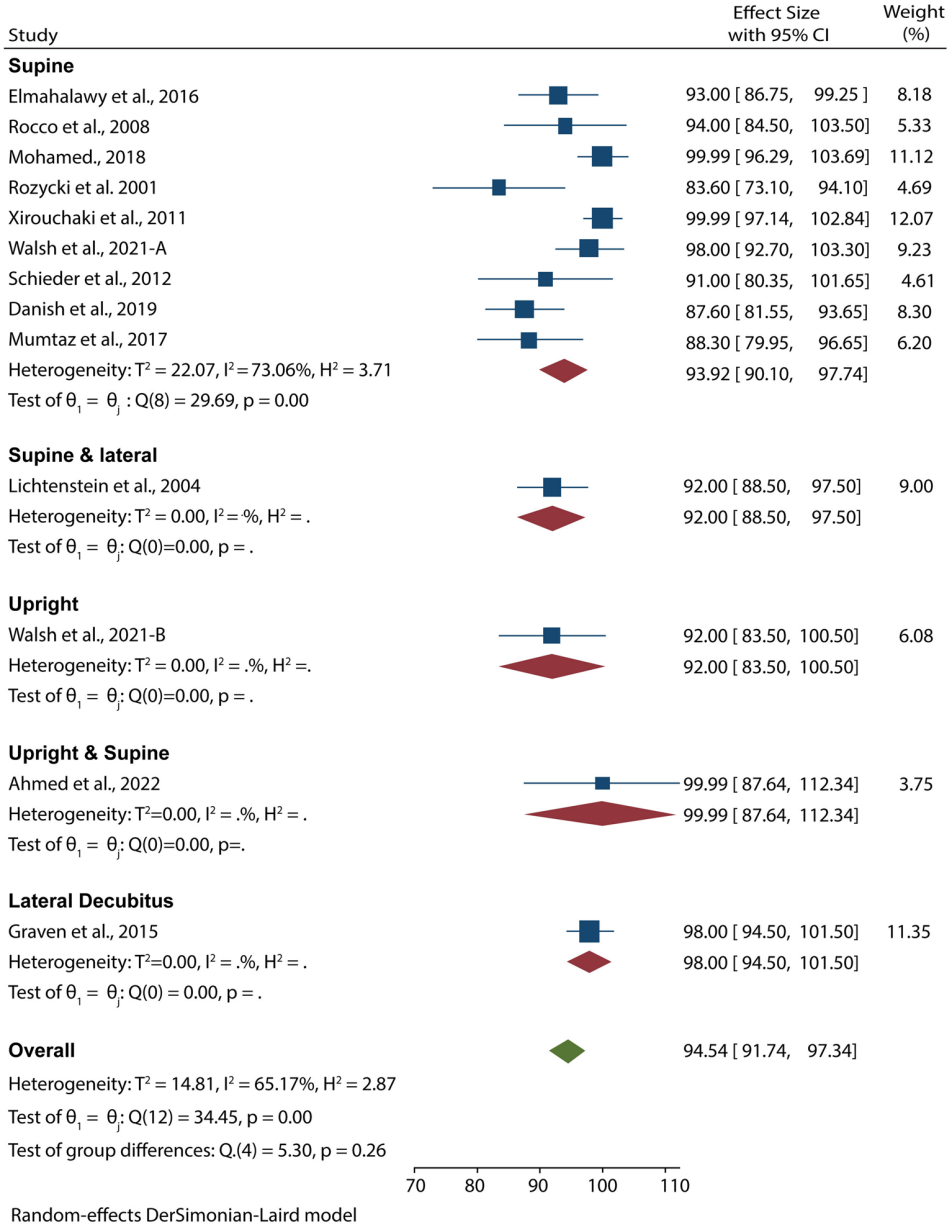
Forest plot showing the sensitivity of POCUS in detecting pleural effusion according to the patients’ position during examination

**Fig. 4:**
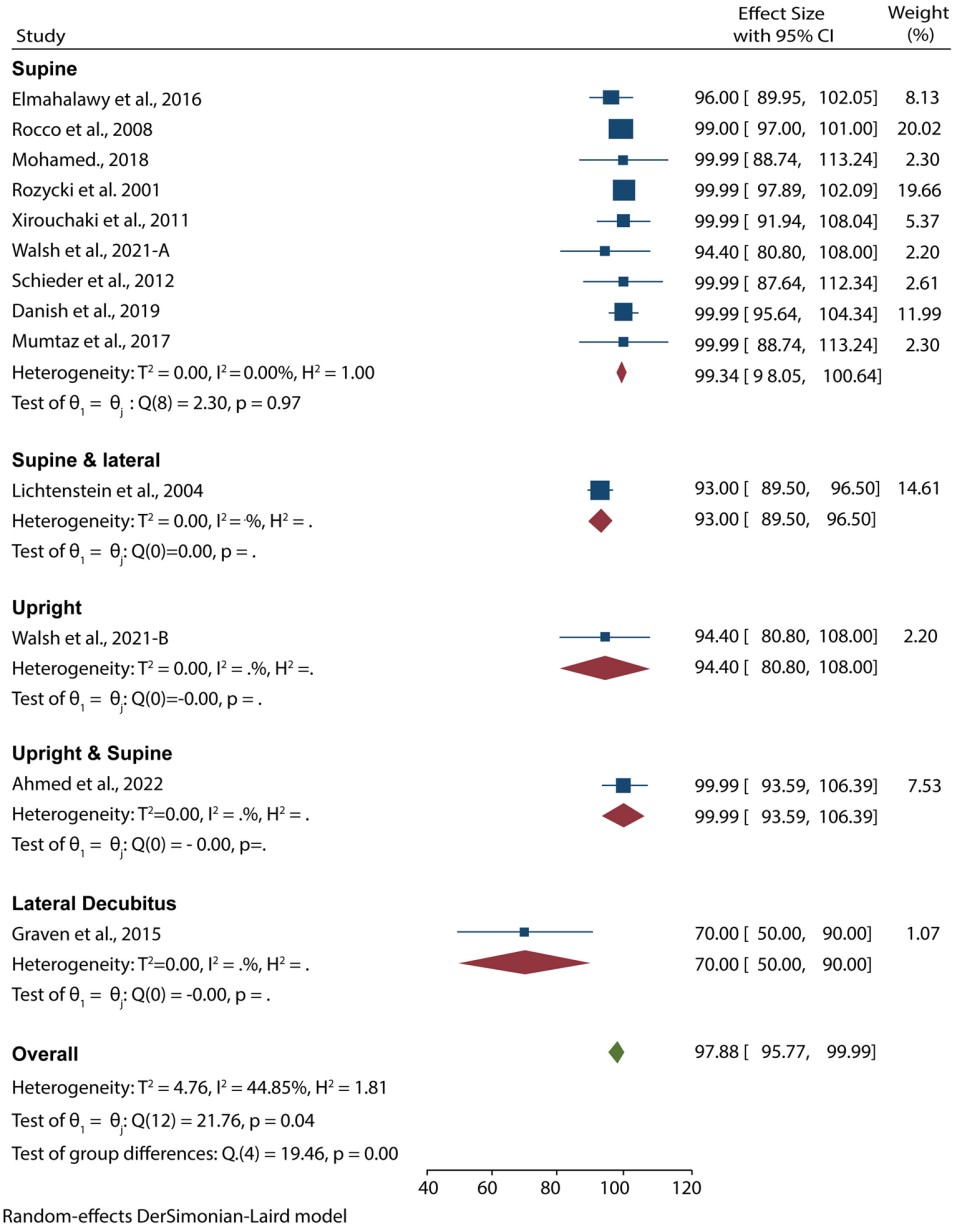
Forest plot showing the specificity of POCUS in detecting pleural effusion according to the patients’ position during examination

12 studies with 653 patients suspected of pleural effusion used POCUS as the imaging diagnostic tool. Data pooled from these studies resulted in an overall sensitivity and specificity of 94.54% (95% CI 91.74–97.34) and 97.88% (95% CI 95.77–99.99), respectively (Figs. 3, 4). Moreover, we carried out a subgroup analysis based on the patient’s position during examinations and found that the test for subgroup analysis on the sensitivity of POCUS was statistically insignificant (*p* = 0.26), suggesting that the position of ultrasound examinations did not influence the sensitivity of POCUS. However, the test for subgroup analysis on the specificity of POCUS was statistically significant (*p* < 0.001), meaning that position during examinations influenced the specificity of POCUS. In these analyses, the specificity was lowest for POCUS exams in the lateral decubitus position (70%) and highest for exams in both supine and upright positions (99%).

### Diagnostic performance of CXR

**Fig. 5:**
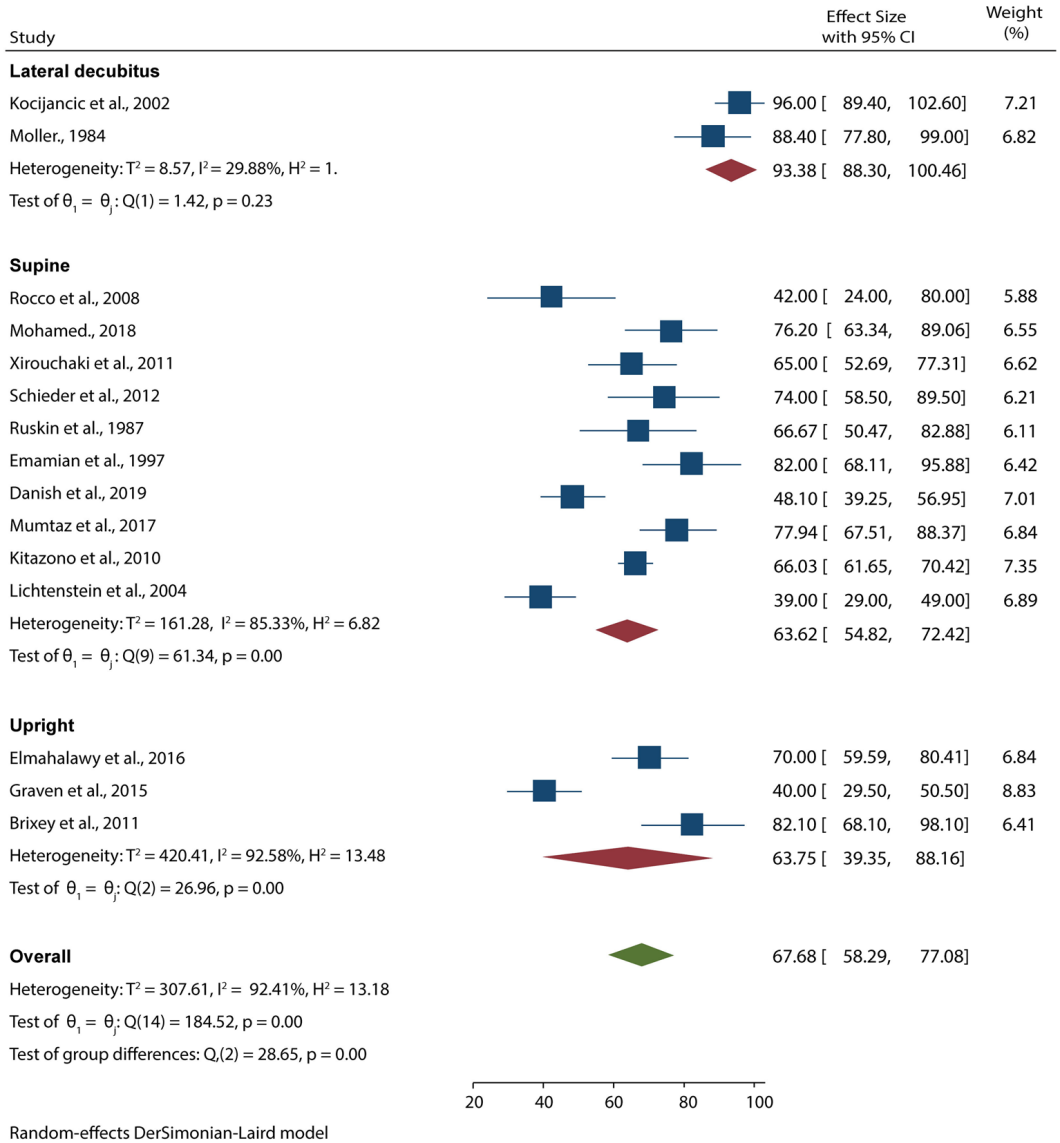
Forest plot showing the sensitivity of CXR in detecting pleural effusion according to the patients’ position during examination

**Fig. 6:**
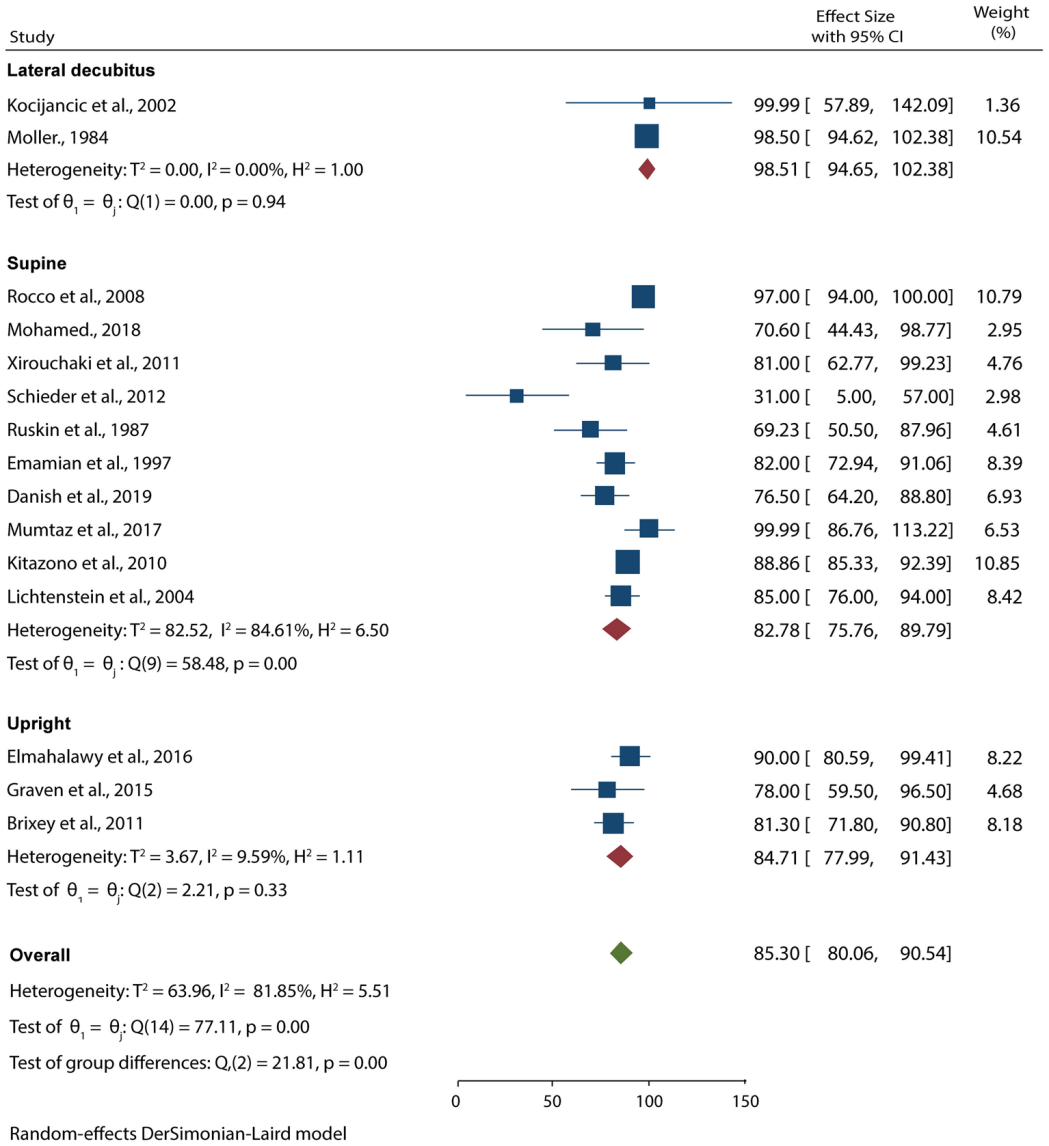
Forest plot showing the specificity of POCUS in detecting pleural effusion according to the patients’ position during examination

15 studies with 955 patients suspected to have pleural effusion used CXR as the imaging diagnostic tool. Data pooled from these studies resulted in 67.68% (95% CI 58.29–77.08) sensitivity and 85.30% (95% CI 80.06–90.54) specificity in detecting pleural effusion (Figs. 5, 6). Our subgroup analysis also showed that the test for differences was significant for both CXR sensitivity (*p* < 0.001) and specificity (*p* < 0.001), meaning that the position of examinations highly influenced the diagnostic accuracy of CXR. From the analyses, we noted that CXR carried out in lateral decubitus position had higher sensitivity and specificity for detecting pleural effusion than supine and upright CXR.

### Subgroup analyses

Further subgroup analyses have shown that the sensitivity of POCUS was higher for procedures carried out in Africa, Europe, and the United States and lower for procedures carried out in Asia. However, none of the other factors, including sample size, machine type, level of training, and reference test, influenced the diagnostic accuracy of this imaging modality. On the other hand, our results have shown that the sample size and reference test influenced the diagnostic accuracy of CXR. The pooled data suggest that the specificity of CXR is higher in studies including 100 patients or more. Additionally, our results suggest that the diagnostic accuracy becomes higher when CXR carried out in other positions is used as the reference test (Table 2).

**Table 2:** Subgroup Analyses of POCUS and CXR diagnostic performance in detecting pleural effusion

Covariate	No. of studies	Sensitivity	Significance	Specificity	Significance
POCUS					
*Sample size*					
≥ 100	1	93 (86.75–99.25)	0.64	96 (89.95–100)	0.54
< 100	11	94.64 (91.66–97.61)		98.03 (95.76–100)	
*Country*					
United States	3	92.20 (84.18–100)	0.02	99.74 (97.68–100)	0.52
Africa	3	97.60 (92.59–100)		98.09 (93.92–100)	
Asia	2	87.84 (82.94–92.74)		99.99 (95.86–100)	
Europe	5	96.46 (93.02–99.89)		95.86 (90.68–100)	
*Level of POCUS training*					
Experienced	10	94.44 (91.20–97.69	0.92	98.25 (96.54–99.97)	0.41
Inexperienced	2	93.92 (84.54–100)		85.97 (56.64–100)	
*Reference test*					
CT scan	8	94.60 (90.73–98.47)	0.92	97.82 (95.56–100)	0.48
Other	4	94.29 (89.74–98.84)		94.99 (87.45–100)	
*Machine type*					
Pocket-size	2	96.38 (90.60–100)	0.51	86.07 (56.75–100)	0.42
Other	10	94.12 (90.80–97.45)		98.25 (96.53–99.97)	
Chest X-ray					
*Sample size*					
≥ 100	3	74.23 (61.22–87.25)	0.37	92.74 (85.41–100)	0.04
< 100	12	65.82 (52.91–78.72)		80.98 (72.79–89.16)	
*Country*					
United States	3	70.40 (60.65–80.14)	0.87	83.04 (73.86–92.22)	0.97
Africa	2	72.45 (64.36–80.55)		84.30 (66.98–100)	
Asia	2	62.89 (33.65–92.13)		88.11 (65.10–100)	
Europe	8	66 (47.70–84.30)		85.48 (77.59–93.36)	
*Operator*					
Radiologist	12	65.73 (52.85–78.61)	0.34	83.15 (76.33–89.98)	0.27
Other	3	73.94 (63.26–84.63)		88.93 (81.24–96.62)	
*Reference test*					
CT scan	9	63.06 (53.93–72.19)	< 0.001	87.79 (82.43–93.15)	< 0.001
CXR	2	93.38 (86.30–100)		98.51 (94.65–100)	
Other	4	65.28 (44.48–86.08)		67.76 (49.58–85.93)	

## Discussion

The current meta-analysis has shown that POCUS has a higher sensitivity and specificity in the diagnosis of pleural effusion than CXR (94.54 vs. 67.68% and 97.88 vs. 85.30%, respectively). When the results were limited to the position of examinations, we noted that the specificity of POCUS can be improved by carrying out the procedure in both upright and supine positions. On the other hand, we noted that CXR performed in the lateral decubitus position has higher diagnostic accuracy than when performed in the supine or upright position.

Our findings are consistent with two previous reviews comparing ultrasonography and CXR in detecting pleural effusion. Yousefifard and colleagues pooled data from 12 studies and found that ultrasonography was 94% sensitive and 98% specific, while CXR was 51% sensitive and 91% specific in diagnosing pleural effusion [26]. Similarly, Grimberg et al. [11] found the sensitivity of ultrasound to be higher than that of CXR in detecting pleural effusion (93 vs. 24%). However, the specificity of CXR was similar to that of ultrasound (100 vs. 96%). The variation in this study can be attributed to the fact the authors included fewer studies in their analyses. Based on these findings, it is safe to say that POCUS is a superior diagnostic tool for detecting pleural effusion than CXR.

Although our findings support the superiority of POCUS, it is evident that CXR carried out in lateral decubitus position has a high sensitivity and specificity. This high diagnostic accuracy can be explained by the fact that lateral decubitus CXR and chest ultrasound are considered more efficient diagnostic tools for detecting small amounts of free pleural fluids [27, 28]. Reports have also shown lateral decubitus CXR is highly sensitive in detecting as little as 50 ml of fluid accumulating in the lungs [29]. Furthermore, the studies used to analyze the diagnostic accuracy of lateral decubitus CXR employed other radiographic findings as their reference test, which affected their results. This is evident from our subgroup analysis which has shown that reference test has a significant impact on the diagnostic accuracy of CXR. Research has also shown that the false negatives and positives in CXR are high, meaning that using it as a reference test is not recommended as some of the diagnoses can be missed and influence the management of pleural effusion.

Our findings also suggest that pocket-size ultrasound devices have lower specificity compared to other ultrasound devices. Additionally, POCUS carried out on Asian patients seems to have a lower sensitivity compared to when it is carried out on patients from other regions. Although there is no definitive reason for these outcomes, we can attribute them to the different study population. For example, Graven et al. [13] included cardiac patients, Danish et al. [23] included patients with acute lung injury score of ≥ 1 and Mumtaz et al. [24] evaluated road traffic patients. Nevertheless, the sample sizes of the studies used in these subgroup analyses was small (< 100). Therefore, further investigation is required to establish the diagnostic accuracy of pocket-size ultrasound devices and determine whether the geographic region of POCUS application might affect its diagnostic accuracy for pleural effusion diagnosis.

Apart from diagnosing pleural effusion, research has also shown that ultrasound examinations can identify the nature of pleural effusion. According to pathogenesis, pleural effusions are categorized as either exudative or transudative. Exudative pleural effusion (EPE) results from inflammatory processes of the pleura and/or decreased lymphatic drainage and is mainly caused by diseases such as pleural tuberculosis and cancer [30, 31], while transudative (TPE) results from the oncotic and hydrostatic pressure imbalances and is mainly caused by systematic factors such as congestive heart failure and cirrhosis. Yang and colleagues investigated the role of high-frequency (3.5, 5.0, and 7.5 MHz) real-time ultrasound in identifying the nature of pleural effusions in 320 patients and found that all 96 patients with TPE exhibited anechoic appearance on the ultrasound exams [32]. On the other hand, of the 224 EPE categorized into non-malignant and malignant, 78 were anechoic, 50 were complex non-septate, 76 were complex septate, and 22 were homogenous. Similarly, Qureshi et al. [33] evaluated the diagnostic accuracy of chest ultrasound in identifying malignant diseases among patients with pleural effusions and noted that it could distinguish between malignant and benign effusions (79% sensitive and 100% specific). In this study, malignancy was associated with a mural or visceral pleura thickness, the presence of visceral pleural nodules, and abnormalities of the diaphragm. It was also reported that ultrasound was capable of revealing the existence of liver metastases. Although these findings show that ultrasound findings can identify EPE and TPE, further studies are required to investigate whether this differentiation is mainly due to sonographic findings alone or combined with other clinical data.

Additionally, POCUS is essential in assessing the volume of pleural effusion, which is important in deciding whether to drain the effusion. Research has revealed that various ultrasound methods have been proposed to estimate pleural fluid accumulation. Roch and colleagues carried out a study to investigate the accuracy of lung ultrasound in predicting pleural effusions of greater than 500 ml in 44 patients on mechanical ventilation [34]. They found a correlation between the interpleural distance measured by ultrasound at the base of the lung or fifth rib space and volume drainage. Additionally, Usta and colleagues measured the maximum distance between the diaphragm mid-height and seated visceral pleura (*D*) and found a strong correlation between D and the expired volume (*V*). Therefore, they derived the equation for estimating the volume of pleural volume as *V* (ml) = 16*D(mm) [35]. Balik et al. [36] also found that there was a strong correlation between pleural volume and the maximum maximal interpleural distance (Sep); therefore, they proposed that the pleural volume can be estimated using the following equation; *V*(ml) = 20*Sep(mm). Despite these proposed estimations, a reliable estimation is still challenging due to various reasons. First, Ultrasound findings are usually affected by the chest cavity size. In taller patients with large chest cavities, the volume of the fluid is normally distributed over a larger area compared to those with smaller chest cavities. Therefore, the amount of fluid in the pleural cavity can be underestimated or overestimated. Secondly, the position of patients during ultrasound exams can influence the distribution of pleural fluid and consequently affect the measurement of the fluid. Third, very large volumes of pleural fluids can influence the measurements due to lung collapse, which causes fluid displacement. Furthermore, visualization of an entire portion of a very large pleural effusion is impossible. Fourth, the shape of fluid accumulation can be affected by the existence of pulmonary solidities. Lastly, research suggests that transverse ultrasound scans tend to overestimate the volume of pleural fluid; thus, a stern standardized ultrasound protocol is required to avoid errors [37].

Unlike CXR, POCUS can also be used to guide the management of pleural effusion. Research has shown that ultrasound-guided thoracentesis is considered the standard care for many patients with pleural effusion in the United States [38]. Moreover, the British Thoracic Society has recommended that all thoracentesis be carried out under ultrasound guidance [39]. Similarly, the American College of Graduate Medical Education requires that pulmonary and critical care professionals are proficient in using ultrasound for thoracentesis [40]. These recommendations have risen from the fact that ultrasound-guided drainage of pleural effusion is increasingly becoming more successful and has low complication rates. For example, a study evaluating site selection using physical exams, CXR, and ultrasound showed that CXR and physical exams resulted in inaccurate site selection in about 15% of patients, while ultrasound prevented accidental organ puncture during thoracentesis in 10% of the cases [41]. Furthermore, it has been reported that the success rate of thoracentesis improves from 66 to 90% when guided by ultrasound [39]. This ultrasound-guided thoracentesis is normally performed using two methods: “site marking” or “direct needle guidance” [42]. In the “site marking” method, the physicians use ultrasound to identify the optimal site and mark it on the skin, after which the thoracentesis is performed without ultrasound. However, when the position of patients is changed, the distribution of fluid changes; therefore, puncture should be performed immediately after site marking. On the other hand, the direct needle guidance method involves observing the correct needle position during puncture in real time and constantly monitoring it. Mayo and colleagues evaluated the safety of ultrasound-guided thoracentesis without real-time visualization and found a very low pneumothorax incidence (1.3%) [43]. Out of the 3 cases of pneumothorax, one resulted from stopcock malposition, while the others resulted either from lung puncture, entrapment, or entrainment of air through the catheter needle assembly. Based on evidence from this study, real-time visualization seems irrelevant during puncture. However, evidence in other studies suggests ultrasound is carried out before and after puncture to assess normal gliding of the lung and to rule out pneumothorax [44]. Furthermore, POCUS can be used to ease and provide safer pleural drainage by guiding the pigtail-type of drainage [45]. Additionally, ultrasound guidance has been found important in managing other conditions. For instance, our previous meta-analysis reported that ultrasound-guided regional anesthesia was superior to parenteral opioids in patients undergoing hip fracture management [46].

## Limitations

The current meta-analysis was also subject to various limitations. First, the study only included studies published in English, meaning that data from studies published in other languages were excluded from our analysis, thus limiting our meta-analysis outcome. Second, our meta-analyses have shown high heterogeneity values. However, this heterogeneity was addressed by carrying out further subgroup analyses, and the fact that most of the studies were of good methodological quality meant that the heterogeneity did not influence the findings of our meta-analyses. Thirdly, most of the studies in this review included small populations (< 100), meaning they had a small sample size bias which may have been transferred to our analyses. Finally, it is difficult to derive the incidences of false negatives and positives between CXR and POCUS from our study because very few studies reported these values to carry out an analysis. Moreover, we did not carry out a subgroup analysis based on the sizes of pleural effusions; therefore, the results presented in this study are general and not for only small or large pleural effusions.

## Conclusion

In summary, our study has found that POCUS has a higher diagnostic value in detecting pleural effusion than CXR. Therefore, considering that POCUS is non-invasive, quick, and can repeatedly be performed at the patients’ bedside, we encourage that it is considered the first-line diagnostic tool for patients presenting signs of pleural effusion. This recommendation is further reinforced by the fact that our results have shown the diagnostic accuracy for POCUS is still very high even with physicians having less training. Moreover, the specificity of this diagnostic tool can be improved by carrying out the examinations in both upright and supine positions.

## Data Availability

All data and materials available online or included in the manuscript.
